# Factors Affecting Quality of Care in Maternal and Child Health in Timor-Leste: A Scoping Review

**DOI:** 10.1177/11786329221110052

**Published:** 2022-07-04

**Authors:** Mahmuda Shayema Khorshed, David Lindsay, Marie McAuliffe, Caryn West, Kayli Wild

**Affiliations:** 1 College of Healthcare Sciences, Division of Tropical Health and Medicine, James Cook University, Townsville, QLD, Australia; 2 College of Healthcare Sciences, Division of Tropical Health and Medicine, James Cook University, Cairns, QLD, Australia; 3 Judith Lumley Centre & Institute for Human Security and Social Change, La Trobe University, Melbourne, VIC, Australia

**Keywords:** Quality improvement, Timor-Leste, maternal health, child health, health services, quality of care

## Abstract

Timor-Leste faces many challenges implementing quality maternal, newborn and child health (MNCH) services due to resource constraints and socio-cultural factors that disproportionately affect the health of women and children. A scoping review was conducted to map the quality of MNCH services against WHO quality standards on: 1. Provision of care, 2. Experiences of care, and 3. Cross-cutting standards. The literature search identified 1058 citations, from which 28 full-text articles met the inclusion criteria. The findings highlight health workers’ limited capacity to provide quality services and referrals. The major reasons for this are: a lack of essential supplies, poor infrastructure and transport, limited opportunities for ongoing learning, and gaps in health information systems. Provision of care standards and cross-cutting standards require attention at a broad systems level. Findings related to experiences of care highlight the importance of effective communication, respect, and emotional support, particularly for vulnerable women and children who have difficulty accessing services, and for those who have experienced violence. These experience-related standards could be addressed at an individual health worker and health service level, as well as at a systems level. This review provides direction to focus quality-improvement initiatives within local health facilities, as well as at municipal and national level.

## Background

Health care for women and children has been an important focus of the Sustainable Development Goals (SDGs). This focus is encapsulated in goal number 3: “Ensure healthy lives and promote well-being for all at all ages.”^1^ The goal outlines specific targets including, to reduce maternal, neonatal, and child mortality (by 2030), to ensure universal access to sexual and reproductive health care services, and to achieve universal health coverage (UHC). All these targets require the coordination and delivery of quality health services within accountable health systems. Health systems that have the capacity to measure and use data to improve services.^2^ Despite substantial progress during the era of the Millennium Development Goals (MDGs), inadequate resources remain a significant challenge to achieving the SDGs.^3^ In regions such as Sub-Saharan Africa and Southern Asia lack of access to quality health care and extreme poverty are major contributory factors to high rates of maternal, neonatal, and child mortality.^4,5^

The World Health Organization (WHO) has widely advocated for improvements in the quality of maternal, newborn, and child health. It has established frameworks and standards for improving the quality of maternal and newborn care, and that of children and adolescents, in health facilities.^6,7^ These frameworks identify standards vital to achieving quality improvement, including: 1. Evidence-based practices for routine care and management of complications; 2. Actionable information systems; 3. Functional referral systems; 4. Effective communication; 5. Respect and preservation of dignity; 6. Emotional support; 7. Competent, motivated human resources, and 8. Availability of essential physical resources. The first 3 standards reflect “provision of care,” the next 3 the “experience of care” and the last 2 address cross-cutting factors which are pre-requisite standards for providing both the provision and experience of care standards.^6,7^

Providing quality health services has proved difficult in low- and middle-income countries.^8-10^ A task made more challenging when resource limitations are combined with ongoing political and social conflict.^11,12^ Timor-Leste is a post-conflict country situated between South East Asia, Australia and the Pacific; it is the newest nation in Asia. Independence from Indonesia was achieved in 2002 after years of occupation.^13^ Substantial progress has been made to rebuild the health care system following Indonesian forces exit from the country, however major challenges remain to achieve health and wellbeing for the nation’s most vulnerable.^14^ The 72% of Timor-Leste’s population live in rural and remote areas and 42% live below the poverty line.^15,16^ The pregnancy-related mortality ratio is 218 deaths per 100 000 live births and infant and under-5 mortality rates are 30 and 41 deaths per 1000 live births, respectively.^15,16^ Maternal and child health services are provided by a multidisciplinary cadre of health workers including doctors, nurses, and midwives.^16^ Delivering quality and accessible maternal, newborn, and child health (MNCH) care remains a priority focus for the Government of Timor-Leste and its development partners.

The aim of this review is to search the literature on MNCH service delivery in Timor-Leste and to map factors affecting quality of care based on WHO quality standards. The review offers a framework for stakeholders who want to improve the quality of care in Timor-Leste, and provides a baseline analysis for designing MNCH quality improvement initiatives linked to WHO evidence-based standards.

## Methods

This review was undertaken as part of a larger study on quality improvement of MNCH services in Timor-Leste. A scoping review methodology was chosen to assess the extent of the literature and to explore factors affecting the quality of MNCH services in Timor-Leste. Arksey and O’Malley’s framework was used to guide the review using the following steps: 1. Identifying the research question, 2. Identifying relevant studies, 3. Study selection, 4. Charting the data, and 5. Collating, summarizing and reporting the results.^17,18^ The review focused on articles published within 5 years prior to commencement of the study in 2018.

## Identifying the Research Question

There were 2 main research questions:

1. What quality oriented MNCH literature exists in the context of Timor-Leste?
2. To what extent do the retrieved articles address the recent WHO quality standards?

## Identifying Relevant Studies

We accessed 7 electronic databases: MEDLINE, Ovid Emcare, Cochrane Library, Cumulative Index to Nursing and Allied Health Literature (CINAHL), PsycINFO, Informit, and Scopus. Gray literature sources, including Google Scholar, Google, and One Search, were searched for additional articles relevant to the topic. A targeted search of the Timor-Leste government website was performed via Google to elicit any publicly available government documents or other gray literature.

The search strategy used the following MeSH subject headings: “Quality improvement” OR “Patient-centered care” AND “Maternal health” OR “Child health” AND “Timor-Leste.”

The inclusion criteria were:

- Published literature
- Unpublished gray literature
- Between 2014 and 2018
- Written in English
- Timor-Leste context
- Relevant to MNCH service delivery

The exclusion criteria were:

- Articles outside of year range
- Languages other than English
- Global or regional reports with minimal Timor-Leste content
- Articles with context outside of MNCH
- Articles that could not be linked to any areas of WHO quality improvement framework

## Study Selection

Tricco et al^19^ describe a 2-staged screening process utilizing the PRISMA framework. One author (MK) assessed the retrieved articles against the inclusion and exclusion criteria. Ambiguous articles were resolved by consensus amongst the co-authors. A sub-sample was then checked by the second author (DL) for accuracy.

Through title screening, articles were excluded that did not relate to quality of health care or MNCH in Timor-Leste. When abstract screening, the keywords “Quality,” “Timor,” “Maternal,” and “Child” were used to assess eligibility. In cases where these words were not identified in an abstract the full text was searched. Articles that described Timor-Leste within a global context or with other countries were included only if they provided a sufficient level of information related to Timor-Leste. While screening full texts, articles were further investigated to assess whether they could inform 1 or more of the WHO quality standards.

**Figure 1.**
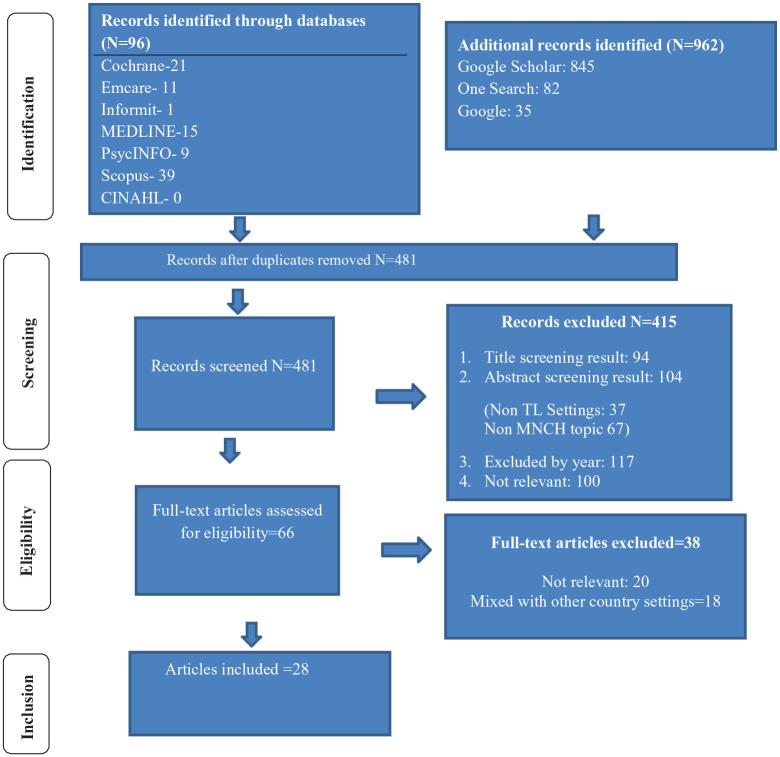
Search summary using PRISMA flow diagram.

The literature search yielded 1058 articles which, after removal of duplicates, was reduced to 481. Four hundred fifteen publications were excluded after title and abstract review because they did not meet the criteria for publication year, Timor-Leste setting, MNCH topic or were assessed as not relevant. Sixty-six full text publications were screened and a further 38 articles excluded because they did not provide relevant information related to any of the WHO quality standards, or because they were multi-country publications that mentioned Timor-Leste but did not provide detailed, country-specific information. Thus, the screening yielded 28 articles for inclusion in the scoping review (Figure 1).

## Charting the Data

A data extraction table for charting study characteristics, key findings, and relationship with WHO quality standards was developed by the authors through consensus. The extraction table consisted of columns to record information on the author, year of publication, objective, study design/type of report, location/study context, sample/participants, article findings, and relationship to WHO quality standards (Table 1). Data were extracted from the full text articles by the first author (MK) and a sub-sample were reviewed by 2 co-authors (DL, KW) for accuracy. Any discrepancy in interpretation was discussed and resolved by consensus amongst the authors.

**Table 1.** Summary analysis of retrieved articles.

No.	Author	Year/Objective	Study design/ Type of report	Location	Sample details	Article findings	Relationship to WHO quality standards
1	Asante et al^51^	2014/To identify the challenges and opportunities for rural retention of health workers in Timor-Leste	Policy paper	Timor-Leste	N/A	The article findings relate to inadequate skilled health workers, improper distribution and their retention in rural areas. Factors which were important were salary structure, trainings, motivation, career development plan.	The article has a dedicated focus on developing a skilled work force in Timor-Leste. This fits with the WHO quality standard of “Competent motivated human resources”
2	Dyer^31^	2015/To oversee the women’s experiences as participants of an m-Health program	Cross-sectional qualitative study	Timor-Leste (Manufahi)	27 in-depth, semi-structured interviews from women past participants in the Liga Inan mHealth program	Women in Timor-Leste face communication and transport challenges for accessing healthcare facilities in emergency situations. The paper looked into effect of Liga Inan, an M-health program on healthy behavioral practices of women.	Women talked about the usefulness of information that they received. Some of them was related to knowledge and practice of preventive healthy behaviors and reaching facility on time for their health checkups. This article is related to WHO quality standards of “Effective Communication” and “Functional referral systems”
3	Hodgins and D’Agostino^25^	2014/To estimate coverage for specific elements of antenatal care	Secondary analysis using Demographic and Health Survey (DHS) data from	41 countries including Timor-Leste	Representative women sample for DHS survey from each country	The article described antenatal care elements independently amongst the pregnant women. Timor-Leste performed average amongst 41 countries having a 42% quality—coverage gap	Quality focuses not only on number of antenatal visits but also toward the elements of care provided during the visit. The article refers to WHO quality standard of evidence-based practice for routine care and management of complications
4	Hou et al^50^	2016/To understand the labor market dynamics among health workers, including their preferences and concerns, and to assess the skills, competence, and performance (ie, the “know-do gap”) of doctors working in Timor-Leste	Cross-sectional survey	Timor-Leste (13 districts)	443 health workers from 69 health facilities, including 175 doctors,150 nurses, and 118 midwives	The study looked into factors affecting understanding, skills and performance amongst different level of health workers. Increased skill and performance, improved working condition for the rural health workforce has been identified as a key to the quality of care. Providers talked on motivation, learning opportunities, supervision, salaries. Nurses and midwives reported on inadequate transportation, nurses also reported on inadequate salaries.	The article critically outlined on Health workers’ confidence, attitudes, skills and has a relevancy to WHO quality standard “Competent motivated human resources”
5	Khanal et al^36^	2014/To identify factors associated with exclusive breastfeeding in Timor-Leste	Secondary data analysis from Timor-Leste Demographic and Health Survey (TLDHS) 2009 to 2010	Timor-Leste (13 districts)	975 infants	Almost half of infants were exclusively breastfed, but the prevalence for exclusive breastfeeding decreased with increasing infant age. Women who were financially solvent and empowered to carry on their decisions could breastfeed exclusively their infants.	Communication was identified the key solution which can be imposed through several approaches for improving breastfeeding practices including antenatal counseling, breastfeeding promotion programs, counseling during home visits etc. Therefore, the article has a relevancy to “Effective Communication” standard
6	Khanal et al^37^	2015/To examine the prevalence and factors associated with under-utilization of antenatal services in Timor-Leste	Secondary data analysis from TLDHS 2009 to 2010	Timor-Leste (13 districts)	5895 mothers	Half of the mothers did not attend health facility for recommended 4 antenatal visits. Mother’s decision-making power is a significant contributor of health service utilization which are often related to their educational background, occupation, or wealth status. Findings indicated need of educating the family/husbands whose possible dominant role are crucial in patriarchal society of Timor-Leste.	“Effective Communication” was the relevant identified standard. The article found communication requires being established with women and/or her family. Antenatal visits maybe used as an opportunity to support them for preparing a birth preparedness plan.
7	Moores and de Jesus^30^	2018/To evaluate prehospital treatment of post-partum hemorrhage (PPH) patients transported by the Timor-Leste National Ambulance Service (TLNAS)	A retrospective audit of PPH Patient-care record	Timor-Leste (13 districts)	214 PPH patients	The articles findings suggest that there is inconsistent PPH management practice by midwives. The underlying challenges are equipment scarcity, inadequate skills, and unavailable PPH is improve clinical practice guidelines for PPH management,	The paper highlighted on need of skill building of providers and resource availability matching with WHO quality standards: 1. “Competent motivated human resources,” 2. “Essential physical resources available,” and 3. “Functional referral system”
8	Nie et al^38^	2016/To assess the level of knowledge, practices, and health service of mother’s coverage related	Secondary data analysis from a baseline survey to collect information about mobile phone ownership and usage patterns	Timor-Leste (Manufahi and Ainaro)	581 women 15-49 y old with a child up to 24 mo of age	The findings reveal those women having a mobile phone more likely to maternal and newborn health services. Interventions such as forming women’s group are required to be targeted to those women who did not have a mobile phone and were more likely to not avail health services. Parallel to those referral care system should be strengthened for ensuring quality services	WHO quality standard of “Effective Communication” is directly related to this article. Engaging the women group with suitable communication strategy is a key to access the healthcare in emergency
9	Provo et al^20^	2017/To evaluate the current nutrition-specific programs in Timor-Leste provide an overview of the country’s “nutrition system” for stakeholders	Assessment report of quality of nutrition interventions delivered through antenatal and postnatal care	Timor-Leste	N/A	The report identified several challenges in providing quality nutrition interventions at the time of providing antenatal and postnatal services in health facilities. There is often delayed care seeking, stockouts of commodities, inadequate capacity of health workers to implement nutritional interventions. Moreover, there is absence of appropriate nutritional interventions; again, when interventions exist, the quality of nutritional interventions is not ensured always: including those provided during antenatal and postnatal services.	The article highlighted on strengthening skill and motivation of health workers to be able to support nutritional interventions targeted to maternal and child groups. The following WHO quality standards were reflected in the article: 1. “Competent motivated human resources,” 2. “Evidence-based practice for routine care and management of complications” 3. “Essential physical resources available”
10	Quinn et al^21^	2014/ To describe infectious disease and health security in Timor-Leste and compare it to state stability status, as ranked on the Failed States Index (FSI).	State case study approach	Timor-Leste	N/A	The paper systematically reviewed Timor-Leste’s post-conflict health system. It identified challenges for health policy in Timor-Leste and mentioned critical areas such as, growing disease burden, malnutrition, inadequate access to healthcare and basic hygiene facilities, gender-based violence, inadequate health infrastructure, inadequate and unskilled health workforce. Ministry of Health, Timor-Leste introduced Servisu Integradu da Saúde Communitária (Integrated Community Health Services or SISCa) that supports disease prevention, early treatment, community awareness, and improve health security.	Solution for tackling the growing disease burden comes through effective implementation strategies including vaccination campaign. A country with a post-conflict experience requires improvement in many basic indicators including that of the “quality of healthcare.” Here the article relates to the below WHO quality of care standards:1. “Evidence-based practice for routine care and management of complications”—which has to be implemented for managing the disease burden. 2. “Essential physical resources available”—Using available resource to ensure required infrastructure and workforce in place 3. “Effective Communication” for building awareness and good practices in community by engaging the SISCa/providers until grass root level
11	Rees et al^39^	2017/To learn about the causes of intimate partner violence (IPV) including the stress of bride price obligations.	Qualitative (in-depth interview)	Dili	1672 pregnant women	The article finds the link between the bride price stress and intimate partner violence among the pregnant women group. One-fourth women who participated in the study reported severe forms of IPV. The article highlighted importance of designing interventions for reducing risk of bride price stress and poverty as a response to IPV.	Pregnant women’s mental health is an important aspect to consider as a quality healthcare component. Women’s emotional turmoil can be overcome by good communication. The topic covers the following WHO quality standards: 1. Effective Communication’, 2. “Respect and preservation of dignity,” 3. “Emotional support”
12	Silove et al^40^	2015/To examine factors specific to a post-conflict LMIC that contribute to perinatal depression and related mental health indices.	Cross-sectional study	Aileu and Liquiça	427 women in the mid-trimester of pregnancy and 3-6 mo post-partum	Over two-fifths of women (186, 43.6%) were encountered within explosive anger category and those faced intimate partner violence are more prone to it. The explosive anger was associated with experiences of mass conflict, financial hardship and physical violence	Experience of care of the vulnerable women group requires to be tackled through “Effective communication” related strategies directed to women and her family that address the sensitive issues. This refers to protecting rights, dignity of women, and preserving the emotional health as described in WHO quality standards:1. “Effective Communication,”2. “Respect and preservation of dignity,” and 3. “Emotional support”
13	Silove et al^41^	2015/To examine perinatal depressive symptoms in women and its associated relation with the post-traumatic stress disorder (PTSD) symptoms	Cross-sectional study	Aileu and Liquiça	427 women in the mid-trimester of pregnancy and 3-6 mo post-partum	Pregnant and postnatal women who experienced intimate partner violence and conflict-related deprivations (murder witnessing, human right violation related trauma) were found to have depressive symptoms	Care during pregnancy and postnatal period in a women’s healthcare cycle has a significant impact on its outcome. Identification and management of post-traumatic stress disorder (PTSD) symptoms may reduce the burden of perinatal depression. “Effective communication,” “Respect and preservation of dignity,” and “Emotional support” are the three key WHO quality standards in this regard
14	Silove et al^42^	2016/To assess the patterns of separation anxiety symptoms amongst pregnant women	Cross-sectional study	Dili	1672 women attending in antenatal clinic	Pregnant women suffered from varied adult separation anxiety (ASA) symptoms were classified in 3 groups: a core ASA (4%), a limited ASA (25%), and a low symptom class (61%). The core group reported to suffer from various trauma and intimate partner violence. They also reported comorbidity with post-traumatic stress disorder (PTSD).	These pregnant women require to be well communicated and provided with emotional support. WHO quality standards of “Effective Communication,” “Respect and preservation of dignity,” and “Emotional support” are related to it
15	Taft et al^43^	2015/Determine the differences in reproductive health and infant and child mortality and health between abused and non-abused ever-married woman in Timor-Leste	Secondary data analysis from TLDHS 2009 to 2010	National	1959 ever-married women aged 15-49 y	The paper identified that ever-married women are at greater risk of violence compared to the never-married women, both in terms of physical or combined physical-mental violence. This results in poor reproductive/infant and child health leading to morbidity and mortality	Women and children’s health outcomes may be improved by preventing violence against women. Communication to the women, her family is important referring to relevant WHO quality standards on experience of care as follows: 1. “Effective Communication,” 2. “Respect and preservation of dignity,” and 3. “Emotional support”
16	Wallace et al^26^	2018/This study aimed to identify factors that influences women’s decisions to seek antenatal care and care during labor and birth in Timor-Leste, a low-middle income newly independent nation in South East Asia with a high maternal death rate	Qualitative	Viqueque, Baucau, Ermera and Dili m	Nine FGDs with 80 men and 17 interviews with reproductive aged women	Associated factors for seeking antenatal care were: role of spouse, birth preparedness, transportation expenses, pregnant women’s own views, and access to healthcare. Maternal provider’s role and status of physical environment were other major factors (adequate water, sanitation, waste management, and energy supply), available logistics and medicines	The identified factors in this article relates elements from WHO quality standards as follows: 1. “Competent motivated human resources,” 2. “Evidence-based practice for routine care and management of complications,” 3. “Effective Communication,” “Essential physical resources available”
17	Wild et al^52^	2015/To understand the role of context, policy characteristics, individual actors, and how evidence is used to influence the policy agenda.	Ethnographic case study	Timor-Leste (National level)	31 senior policy-makers and stakeholders	The paper describes policy level engagement and support for maternity waiting homes. It found that evidence-based policy is supported by the connection between research and policy-makers.	“Essential physical resources available” is the identified WHO quality standard-While maternity waiting homes were thought to be a logical solution to the problem of access and remoteness by policy-makers, they did not address underlying requirements that were absent in many areas (quality birthing facilities, electricity, running water, and emergency obstetric care).
18	Wild et al^44^	2019/To understand the knowledge and needs of midwives in responding to violence against women	Qualitative interviews and focus group discussions (FGDs	Dili, Baucau and Liquica	36 midwives, 12 community participants	Midwives had reasonable knowledge on the fact that women’s mental and physical health is impacted by violence and associated stress. They admitted that affected women fear on opening up. They acknowledge that they needed to intervene with medical treatment and counseling.	The topic of violence itself is a sensitive one and can only be tackled with skills, empathy and smart communication. The report highlighted importance of improving midwives’ skill, knowledge; creating an enabling environment to support their work, Therefore, it covers several quality standards: “Emotional support,” “Respect and preservation of dignity,” “Effective Communication,” and “Competent motivated human resources”
19	Wilkins et al^27^	2015/To identify rate, timing and causes of stillbirths in the National Hospital Guido Valadares, Timor-Leste	Retrospective record review of hospital birth registry and maternal records	Dili	5304 births	The study identified scarcity of data, poor and missing data in National Hospital, which is a barrier for providing quality healthcare services. The article indicated skill building need for providers at the subnational level from where huge number of cases are referred to national level.	Most stillbirths occurred in the antenatal period, emphasizing the need for improved education and awareness among pregnant women and antenatal care providers about fetal movements and other danger signs. Therefore skill building of providers on antenatal services is crucial. Through effective communication and knowledge development, pregnant women, and their family maybe educated. Related WHO quality standards are: 1. “Competent motivated human resources,” 2. ‘Evidence-based practice for routine care and management of complications.3. “Functional referral systems” and “Actionable information system”
20	Zin et al^23^	2014/To understand island health issues within the western Pacific context	Review of country specific health data and relevant literature	Pacific countries including Timor-Leste	N/A	The article identified critical disease burden in island countries leading to higher mortality. Maternal and antennal care was identified as a priority. Other key areas identified were health workforce, and control of communicable and non-communicable diseases	Importance of evidence-based care and skilled workforce has been highlighted in this article. These are linked to the below WHO quality standards “Competent motivated human resources,” and ‘Evidence-based practice for routine care and management of complications.
21	Yeates and Pillinger^49^	2018/To examine international policy responses to cross-border health worker migration in the Asia Pacific region	Review of international datasets and secondary data	Asia pacific countries including Timor-Leste	N/A	The document explained situation of health work force in Asia pacific region that includes Timor-Leste. It explained that the high maternal mortality ratio in TL is associated with low worker—population density and low health expenditure. There is critical shortage of skilled birth attendant. The article pointed to importance of skilled HR, geographic distribution of health providers and their competency as measures of high-quality healthcare services	Health workers are central element of Maternal, Newborn and Child Health (MNCH) health services in Timor-Leste. The WHO quality standard “Competent motivated human resources” matched with the theme of article.
22	Republica Democratica De Timor-Leste^33^	2014/To outline plan and progress for achieving the MDGs in Timor-Leste	TL Government report	Timor-Leste	N/A	In this article, maternal and newborn health (MNH) service challenges are revealed. Maternal health challenges are: data availability, accessibility to healthcare services, knowledge, and awareness on healthcare services. Child health services were improved owing to improved access to health facilities, available supplies and medicine, educational campaign to aware mothers on child health so that they can respond appropriately	Several factors on MNH health services are identified in this article that associate with below WHO quality standards: “Effective communication,” “Competent motivated human resource,” “Essential physical resources available,” “Actionable information system”
23	UNICEF^28^	2015/To provide an overview of children’s rights to health and nutrition, water, sanitation and hygiene, education, protection, and participation, with a special focus on disadvantaged children and their families	Situation analysis report	Timor-Leste	N/A	The report documented some of the key quality elements of child healthcare services: essential medicines in place, fuels for referral; data on child healthcare services, skilled provider, and use of available interventions.	Four WHO quality standards fit with the articles: “Essential physical resources available,” “Evidence-based practice for routine care and management of complications,” “Competent motivated human resources,” and “Actionable information system”
24	General Directorate of Statistics, Ministry of Planning and Finance and Ministry of Health^16^	2018/To provide data for monitoring the population and health situation including that of MNCH in Timor-Leste	Demographic health survey report	Timor-Leste	12 607 women (15-49), 4622 men (15-59)	The report shows improvement of several MNCH indicators. Maternal morbidity and mortality indicators, timing and quality of antenatal care were described which provides a basis for understanding the healthcare quality issues. The report also provided data on child immunization, child- hood illnesses	The report focuses on MNCH healthcare service areas and links to WHO quality standard as the followings: 1. “Evidence-based practice for routine care and management of complications” and 2. “Competent motivated human resources”
25	Republica Democratica De Timor-Leste^22^	2018/To summarize a 5-year proposed government policy plan on 5 key sectors, 1 being the sector of social capital development which includes health as 1 of the sub-categories	TL Government report	Timor-Leste	N/A	The document included TL government’ focus for general improvement of health infrastructure, human resource; logistic and supplies availability, access to health information system, improved referral system. The plan also included MNH program priorities including increased rate of skilled attendance at birth, coverage of antenatal and postnatal health services, improving nutritional status of the children, ensure routine immunizations.	In general, the document covered the below WHO standards: 1. “Competent motivated human resources,” 2. “Evidence-based practice for routine care and management of complications,”3. “Essential physical resources available”
26	World Health Organization^32^	2016/ To consult on the Health Care Quality Improvement Network in the Asia-Pacific Region	WHO Meeting report	Cambodia, China, Fiji, Australia, Japan, republic of Korea, Singapore, Solomon Islands, Papua New Guinea, New Zealand, Mongolia, Malaysia, Lao PDR, India, Sri Lanka, Nepal, Maldives, Bhutan, Myanmar, Thailand, Timor-Leste	Representatives of 21 countries from Asia pacific region, experts from WHO and OECD.	Participants updated on quality improvement initiatives and informed on success factors for improved MNCH/overall services in respective countries. The challenges for healthcare quality in Timor-Leste were presented as follows: Poor record keeping and reporting system, inadequate health infrastructure, absence of audits and accreditation, inadequate training and dissemination activities on QI, poor communication etc.	The meeting report provided an excellent comparison of quality-of-care implementation in several countries of Asia pacific region including Timor-Leste. Some of the key elements found in the report which were comparable to WHO quality standards mentioned below: “Essential physical resources available,” “Effective Communication,” “Actionable information system”
27	World Health Organizatio n^29^	2017/To provide an analysis of current status of baby-friendly hospital initiative	WHO situation analysis report	117 member states including Timor-Leste	N/A	The report findings confirm Timor-Leste’s integration of ten Steps to successful breastfeeding into national quality standards for maternal, newborn and child healthcare and national policies, strategies. Challenges lie in addressing the shortage of providers, maintaining provider’s skill and having a good monitoring system to ensure HW’s performances.	The successful implementation of BFHI program requires human resource engagement implementing the policy and strategies. Two important WHO quality standards are recognized in this regard: “Evidence-based practice for routine care and management of complications,” “Competent motivated human resources”
28	World Health Organizatio n^24^	2016/To provide a summary of Technical Advisory Group meeting on Immunization and Vaccine-preventable Diseases	WHO Meeting report	Western pacific countries	Seven TAG members, 6 temporary advisers, 28 participants from 16 countries and areas, and 76 representatives from partner organizations, and WHO staff from headquarters, the Regional Office for the Western Pacific and representative country offices.	Important quality improvement relevant issues came up in the report: vaccine safety and regulation, decision making process of new vaccine introduction, equitable distribution of vaccines; maintain available stocks, strengthen vaccination record keeping and reporting. The unique challenges for a weak health system include inadequate and unskilled health workers, insufficient cold chain capacity, poor technical, and management capacity	Western Pacific Region has a focus on immunization program to combat the vaccine-preventable diseases. The article found out the following WHO quality standards related to immunization program in the article: “Evidence-based practice for routine care and management of complications,” “Actionable information system,” “Essential physical resources available”

## Collating, Summarizing, and Reporting the Results

The findings from the retrieved articles were mapped against each domain of the WHO quality standards to provide a narrative overview of existing literature related to each of the themes. A basic description of the number of articles that fell into each domain was included to provide an overview of the extent and distribution of the studies.

## Findings

The findings section is presented according to the WHO quality of care standards: 1. Provision of care standards, 2. Experience of care standards, and 3. Cross-cutting standards. A total of 28 articles were included in the review, many of the articles covered multiple standards. Nineteen articles were related to provision of care, 25 articles were related to experience of care, and 24 articles addressed cross-cutting standards (Table 2).

**Table 2.** Number of retrieved articles linked with WHO quality standards.

WHO standards	No. articles addressing the standard	No. articles by sub-category of standards
Provision of care standards	19	Evidence-based practice for routine care and management of complications (11 articles)
		Actionable information system (5 articles)
		Functional referral systems (3 articles)
Experience of care standards	26	Effective Communication (14 articles)
		Respect and preservation of dignity (6 articles)
		Emotional support (6 articles)
Cross-cutting standards	24	Competent motivated human resources (14 articles)
		Essential physical resources available (10 articles)

### Provision of care

The provision of care domain covers 3 elements: a. Evidence-based care practices for routine care and management of complications, b. Referral, and c. Data management. The review findings on this domain are summarized below.

#### Evidence-based care practices for routine care and management of complications (11 articles)

The findings indicate the importance of service providers adhering to clinical protocols in managing nutrition interventions,^20-22^ infectious diseases,^21,23^ immunization,^15,22,24^ antenatal care,^24-27^ and child health services.^28,29^ One report cites the inadequate capacity of health workers, limited availability or stock-outs of commodities, and delayed care-seeking, as challenges to implementation of nutritional interventions within antenatal and postnatal care services in Timor-Leste.^20^ Another article suggests that women will seek health care from a facility when they have confidence in the health care provider, which relies on the provider following evidence-based guidelines.^25^

#### Functional referral system (3 articles)

A functional referral system manages referral cases according to protocols. Rapid transportation to higher-level facilities is used when required. Three articles identified poor road conditions in Timor-Leste as posing difficulties for referral transportation.^27,30,31^ One article contended that high numbers of referrals to tertiary hospitals were due to low skill levels of health care providers in community settings .^27^ Another study described the management of postpartum hemorrhage (PPH) by paramedics while transporting patients to a higher-level facility, using the Timor-Leste National Ambulance Service.^30^ The retrospective audits found paramedics diagnosed 85% of cases, but did not regularly use resources available to them such as oxygen and intravenous isotonic crystalloid fluid. This study illustrates how referral systems are linked to other provision of care standards such as the routine management of complications.

#### Actionable information systems (5 articles)

The review identified gaps in information systems, highlighting improper record keeping practices, missing records, and a scarcity of data, particularly with regard to maternal health, child health, and vaccinations.^24,27,28,32,33^ According to 1 study, more than 60% of death data was found to be missing or incomplete in tertiary hospital settings.^27^

### Experience of care

The experience of care standards refer to the experiences of clients at the point of care.^6,7,34^ The 3 elements covered are: a. Effective communication, b. Respect and preservation of dignity, and c. Emotional support. All these elements are critical in the post-conflict social landscape of Timor-Leste. Several articles in the literature review highlight experience of care through the concept of patient-centered care, a model that prioritizes the needs and rights of the person receiving care and puts them at the center of decisions that affect them.

#### Effective communication (14 articles)

The review identified 14 articles that addressed the theme of “communication” to facilitate healthcare delivery and improve the experiences of pregnant women and their children.^21,26,31-33,35-43^ Two articles recommend antenatal counseling by skilled providers, a provision that can impact women’s decision-making during labor and childbirth and influence breastfeeding practices.^36,37^ Midwives play an important role in the communication of information to women both in-person and by using mobile phone technology.^31^ One article describes the implementation of Liga Inan (Connecting Mothers), an mHealth program designed to promote communication between midwives and pregnant women using mobile phone messaging.^38^ Women described the usefulness of the service, which provided information on healthy behaviors and reminded them when they had an appointment at a health facility. Having access to a mobile phone helped women receive health information and increased their understanding of health care services. One study found a link between mobile phone ownership and increased use of maternal and newborn health services, however after adjusting for socioeconomic factors, mobile phone ownership was not independently associated with service use.^38^

Intimate partner violence (IPV) is common in Timor-Leste. Women who have experienced such violence exhibit higher rates of sexually transmitted infection, low birthweight births, and pregnancy termination than the general population. They have lower attendance at antenatal clinics and are at greater risk of morbidity and mortality.^39^ Health providers can play a critical role in addressing the issue of IPV through sensitive communication with women about their needs, and by providing advice on how they can best be assisted to safety.^39^

Explosive anger,^40^ depression,^41^ and adult separation anxiety (ASA)^42^ have been observed in women, especially married women, during their pregnancy and postnatal period.^43^ If IPV and mental health issues are not addressed there could be significant consequences for women and children’s physical and mental health. In one study, midwives emphasized that, in addition to sensitive communication, women require health system factors like patient privacy and suitable appointment lengths to be in place so that they can talk openly about their problems.^35^

In a global consultation forum poor communication was reported as a barrier to the delivery of quality healthcare services.^32^ One Government report emphasized women’s lack of knowledge about how to access health care services, indicating the need for broader communication with communities and outreach by health services.^33^ The article also highlights the role of educational campaigns in improving people’s understanding of child health issues. For example, Servisu Integrado du Saude Comunidade (SISCa), an Integrated Community Health Service Program introduced by the Ministry of Health, serves as an awareness building platform for the prevention of diseases.^21^

#### Respect and preservation of dignity (6 articles)

Respect and dignity are an essential element of quality of care. Articles from the literature search related to this domain were mostly focused on the needs of women who have a history of trauma and violence.^39-44^ Health providers’ empathy, confidentiality and communication skills, as well as referral of women to appropriate services, play a critical role in increasing patient safety and preservation of dignity.^44^

#### Emotional support (6 articles)

Similar to the need to show respect and preservation of dignity, women who have experienced trauma need significant emotional support.^39-43,45^ One article found that a quarter of study participants were subjected to IPV, which was associated with cultural obligations and bride price stress.^39^ Midwives providing health services to women acknowledged the fear felt by women speaking out about the violence they face.^45^ The midwives recognize the importance of kindness and emotional support when providing counseling and discussing treatment options with these women.

### Cross-cutting standards

Cross-cutting standards include competent and motivated human resources, and the availability of essential physical resources. Available human and physical resources were the key challenges identified in this review. Inadequate availability of skilled providers, especially in rural settings, has been a key constraint in Timor-Leste since independence.^46^ A study performed in Rivers State, Nigeria found that more than 50% of primary health care workers wanted to move away from their current place of work.^47^ Similarly, rural retention has been identified as a problem in a policy analysis in Bangladesh.^48^

#### Competent motivated human resources (14 articles)

A critical element in delivering quality health care is a skilled workforce, identified in the WHO quality standards as a “competent motivated human resource.” Fourteen articles in the literature search included issues related to human resources for health in Timor-Leste.^16,20,22,23,26-30,33,44,49-51^ Two government reports focus on interventions to do with human resources and identify increasing the number of skilled-birth attendants (SBAs) as a priority program area for MNCH services.^22,33^ Two articles highlight Timor-Leste’s health workforce crisis within the Asia Pacific region and focus on insufficient number of SBAs.^23,49^ The latter article also highlights low worker population density and low expenditure on health services as factors that contributed to high maternal mortality in Timor-Leste.^49^

One article reports additional challenges in providing skilled nutrition interventions to women and children when there is a scarcity of supplies and commodities, or when the women arrive late for care.^20^ Another article stresses that skilled health care workers are required to deliver quality health care services to children^28^ The limited skills of midwives was described as a challenge in 1 article, which found inconsistent practices in the management of post-partum hemorrhage (PPH) when transporting women to health facilities.^30^

The 2016 Timor-Leste Demographic and Health Survey reported that only 57% of births were assisted by skilled health care workers, and that the number of those workers available to assist with births varied substantially by municipality.^16^ Quality antenatal care provision requires the employment of skilled maternal health workers however, less than half (45%) of women received the recommended 4 antenatal visits.^16^ One report described Timor-Leste’s achievement in integrating the 10 Steps to Successful Breastfeeding into national quality standards for MNCH.^29^ Two articles describe the shortage of health care workers as a critical issue, especially in rural areas.^50,51^ Midwives are the core providers managing pregnant women and children in health care facilities and are often required to handle sensitive cases of pregnancy-related violence and/or mental health issues.^45^ They are therefore a central component of the health workforce providing healthcare services to women and children.^16^

Pregnant women reported lack of confidence in the skill of providers as a reason for not visiting health care facilities.^26^ One article attributed the high rate of referrals to tertiary level health facilities to the inadequate skill of health care providers in lower-level facilities.^27^ Health care workers identified inadequate pay, transport difficulties, lack of training or continuous learning opportunities, and inadequate supervision as important barriers to providing quality MNCH services.^50^

#### Essential physical resources available (10 articles)

Timor-Leste has progressed significantly in rebuilding its health infrastructure since it achieved independence from Indonesia,^21,22,32,33^ but more development is needed. Two major challenges for the country are continued improvement of the infrastructure and ensuring widespread availability of essential resources.

Lack of adequate water and sanitation were identified as reasons why women do not seek antenatal care.^26^ Stock-outs of commodities have been identified as a challenge to implementing nutrition^20^and vaccination programs,^24^ making it harder for providers to offer a quality service. Even when skilled health workers are available equipment scarcity poses challenges, for example, when managing PPH patients during transportation via ambulance.^30^ Fuel shortages for ambulances result in a threat to successful critical care outcomes.^28^

One paper analyzed policy-level support for building maternity waiting homes and found that they were considered an appealing policy option because they attract donor investment in infrastructure. There is little evidence to show that waiting homes improve access to care for women in remote areas.^52^

One Government article outlined a 5-year plan for improving logistics, supplies and infrastructure to address quality within health care services.^22^

## Discussion

This review on the state of MNCH care in Timor-Leste found significant limitations across all 3 of the WHO quality standards including the provision and experience of care as well as cross-cutting standards.

## Provision of Care Standards

The review highlights the need for evidence-based guidelines and protocols for managing critical health issues, particularly in maternal and child health, as priority areas for intervention. Evidence-based practice of MNCH was positively influenced by workers’ skill-building through training and support for implementation.^53,54^ A fact that reinforces the need to continue support for developing the knowledge and skills of health workers. In addition to clinical guidelines and training, it is important to include regular refresher training, based on knowledge and skill gaps, and to support implementation with ongoing mentoring within an enabling environment.

Access to health care and timely referral are ongoing challenges in Timor-Leste.^27,30,31^ Challenges associated with maternal and neonatal referrals have been widely reported in other low- and middle-income countries.^55^ Factors influencing referral systems in Timor-Leste are: low skill levels of health care workers in charge of managing complications, lack of staff in health facilities, poor road networks, fuel scarcity and a lack of functioning vehicles, and poor weather conditions—particularly in the wet season. The challenges associated with referral are exacerbated by the difficulties people face accessing health services, which include: lack of transport, poor road conditions, gender inequality, and poverty.^56^ These factors combine to severely limit access to appropriate levels of care, particularly for women and children in remote areas. In the face of these challenges, women and their families tend to rely on traditional medicine and assistance from traditional birth attendants.

In addition to improving referral between levels of service, research from Timor-Leste recommends improving quality of care within health services, increasing the availability and functioning of general patient transport services, and the provision of travel subsidies to patients and their families.^56^

## Experience of Care Standards

Studies related to experience of care appeared the highest number of times in the retrieved articles (total 25 articles). Health providers, especially midwives who provide the majority of maternal, child, and reproductive health care, have a critical role in establishing a respectful and supportive relationship with their clients, communicating information, and discussing care options with women and their families.

In Timor-Leste there are high rates of gender inequality and normalization of violence against women and children, which result in adverse health outcomes.^45^ Other research from Timor-Leste demonstrates the damage domestic violence does to women’s physical and mental health, examples of which include explosive anger and long-term depression.^40-42^ Health workers are often the first service providers women come into contact with as they seek care for injuries or chronic health problems resulting from IPV. In these situations, it is critical that health providers can offer emotional support, safety planning and appropriate referrals, in addition to medical care. However, in Timor-Leste it is common for health providers to blame women and to ask them what they did to cause the abuse.^35,57^ Although there are national guidelines for health providers to assist survivors of IPV,^35^ there is much to be done to improve communication, respect, and emotional support to vulnerable people given the magnitude of violence against women, children, and people with disabilities in Timor-Leste.^58^

## Cross-cutting Standards

The availability of human and essential physical resources in the health sector are key challenges identified in this review. A lack of skilled health care workers in rural settings has been a constraint to health service delivery since Timor-Leste’s independence from Indonesia.^14,46,56^ Factors that contribute to the lack of skilled health care workers in Timor-Leste include: limited opportunities for continuous learning, insufficient supervision, poor working conditions, lack of transport, and low salaries.^50^ A study of health worker migration in Asia Pacific reported that retention of health workers in rural areas is a critical problem that could be addressed through education, personal and professional support, financial incentives, and regulatory and health system supports.^49^

This review also reinforced the need for sufficient physical resources, without which skilled health workers may not be able to provide adequate care. In Timor-Leste, the delivery of MNCH services continues to be hampered by a lack of infrastructure, stock-outs of essential equipment and medicines, poor water and sanitation facilities, a lack of functional ambulances and emergency transport, and a lack of time and privacy during consultations.

## Conclusion

This review has captured recent literature related to WHO quality standards in the delivery of MNCH services in Timor-Leste. As the Ministry of Health and its development partners focus on improving quality of care in Timor-Leste, the findings provide direction on specific areas that can be targeted for MNCH improvement. While many of the issues identified are systemic and require high-level policy and system-wide support (such as evidence-based guidelines, referral systems, health information systems, deployment of human resources, and essential infrastructure and supplies), other issues can be addressed at the provider and health facility level (such as effective communication, respect, emotional support, distribution of clinical resources, and reflection on and use of health system data). There is a growing body of evidence that quality improvement initiatives can be effective in low-resource settings.^59,60^ However, these initiatives rely on accurate information and health system data. Poor quality data was a major challenge identified in this review^24,27,28,32,33^and has been documented in MNCH services in other low-resource settings.^61,62^ An important first step in the quality of care agenda in Timor-Leste is therefore improving case recording, and educating health providers as to its importance. In addition, building skills in data management and the use of information for resource allocation, reflection, and for planning quality improvement initiatives. The country also requires strategies and practical solutions to overcome geographic constraints to accessing care. The challenges facing health services are diverse, and therefore further work is needed to identify specific local needs, strengths, and resources and to consider how this knowledge can be used to improve MNCH services in distinct parts of the country and at various levels of the system. Given the multi-layered and intersecting nature of quality standards, initiatives will need to be addressed in all parts of the health care service, at national and municipal levels, in local health facilities, and by individual health care workers. Stakeholder’s critical understanding of these vital quality standards can enhance policies to improve MNCH services in Timor-Leste. Policy formulation on MNCH quality improvement in Timor-Leste requires critical linkage of matching the existing situation with the available quality standards.
